# Effect of Limestone Powder Fineness on the Physical and Mechanical Performance of Concrete

**DOI:** 10.3390/ma18040835

**Published:** 2025-02-14

**Authors:** Mingming Zhang, Yuanqi Yang, Yuepeng Wu, Jianbo Yu, Haoran Pang, Henglin Lv

**Affiliations:** 1 School of Mechanics and Civil Engineering, China University of Mining and Technology, Xuzhou 221116, China; zmm2020cumt@163.com (M.Z.); dapu0922@163.com (Y.W.); 15965086918@163.com (J.Y.); 18369602248@163.com (H.P.); 2 Jiangsu Collaborative Innovation Center for Building Energy Saving and Construction Technology, Jiangsu Vocational Institute of Architectural Technology, Xuzhou 221116, China; 3 Jiangsu Key Laboratory Environmental Impact and Structural Safety in Engineering, China University of Mining and Technology, Xuzhou 221116, China

**Keywords:** limestone powder, fineness, concrete, physical properties, mechanical properties, compressive activity index

## Abstract

To study the impact of limestone powder (LP) fineness on the physical and mechanical characteristics of concrete, a total of 88 sets of LP concrete mixtures were designed, in which the LP content (0~35% of the mass of cementitious materials), LP-specific surface area (350–1000 m^2^/kg), and water–binder ratio were used as testing factors. The experimental and theoretical approaches were performed to study the physical (slump) and mechanical properties (compressive strength and bond-slip behaviors with steel bars) of LP concrete. Secondly, the compressive activity index (CAI) was introduced as a measurement for quantifying the compressive activity of LP. The results indicate an optimal improvement in the physical and mechanical properties of LP concrete when the LP content is equal to or less than 15% and the specific surface area is equal to or less than 600 m^2^/kg. An increase in LP content will result in a drop in the bond strength between concrete and steel bars; in contrast, an increase in LP-specific surface area will enhance the bond strength. Furthermore, CAI can better reflect the role of LP in concrete, which provides a theoretical basis for the application of LP in concrete.

## 1. Introduction

Civil engineering construction has experienced significant growth in recent years, resulting in a considerable demand for concrete [1,2]. As the most significant cementitious material in concrete, the production process of cement not only consumes substantial resources, including coal, limestone, iron ore, and clay, but also emits considerable amounts of CO_2_, SO_2_, NO_X_, and other gases, which severely pollute the environment [3]. In order to address these issues, auxiliary cementitious materials such as fly ash, slag, and silica fume are commonly utilized to partially replace cement. This approach not only reduces the quantity of cement clinkers required but also conserves energy and resources. It can also fully utilize the waste generated by industrial production, protect the environment, and achieve ‘’green’‘ development in the concrete industry [4,5]. However, the continuous growth in the demand for concrete has made it challenging for the output of conventional mineral admixtures, such as fly ash and slag, to meet current market needs. Additionally, issues such as the uneven geographical distribution of these resources persist [6,7]. In this context, limestone powder (LP) has garnered increasing attention due to its availability, cost-effectiveness, and high yield [8,9]. The use of LP as a concrete admixture not only helps alleviate the shortage of traditional admixtures but also provides significant economic, ecological, and social benefits [10,11,12].

As discussed in previous studies, LP has shown a good water-reducing effect and can effectively improve the working performance of concrete [13,14]. Under the same initial slump state, the increase in LP content will lead to the rise in concrete slump loss, but the correct quantity of incorporation can still maintain satisfactory working performance [15,16]. When the LP content is low (mass fraction of 10%), it can greatly enhance the early and late compressive strength of concrete by filling the gaps between cement particles, optimizing the internal structure, and improving compactness [17,18,19]. When the content of the LP grows to a medium level (mass fraction of 15~20%), the compressive strength of concrete may either remain unaltered or experience a minor reduction. This is because the filling effect of LP on the improvement in the internal structure compactness of concrete begins to weaken, while the strength weakening effect caused by the reduction in hydration products caused by the reduction in cement clinker gradually increases [20,21,22]. With the LP content continuing to rise (reaching a mass fraction of 30% or higher), the compressive strength of concrete will noticeably decline. This decline can be attributed to the fact that the elevated LP content hampers the hydration activity of cement [23,24,25]. At the engineering application level, limestone powder has been extensively utilized in high-fluidity concrete and high-performance shotcrete in Japan since the late twentieth century. The concrete used in the bridge pier and cable anchorage of the Mingshi Strait Suspension Bridge contains 150 kg/m^3^ of limestone powder, which constitutes 36.6% of the total powder material [26]. In Germany, Portland cement II/A-L comprises 80% to 90% clinker and 6% to 20% limestone powder [27]. LP has been extensively utilized in large-scale projects, and relevant specifications have been established to outline specific requirements for its content and performance [28,29,30].

In terms of durability, due to the low activity of LP, the content of hydration products in a unit volume of cement-based materials decreases, which leads to a decrease in strength and freeze–thaw resistance, and this effect is positively correlated with LP content [31,32,33]. For low-strength grade concrete, increasing the content of LP from 0% to 20% can improve the resistance to chloride ion penetration of concrete but will reduce its frost resistance [34]. When the content of limestone powder LP is 5%, the freeze–thaw resistance of concrete is the best; when the content of LP is between 5% and 10%, the frost resistance is higher than that of ordinary concrete; when the content of limestone powder increases to 10~20%, the freeze–thaw resistance of concrete gradually weakens [35]. In order to solve this problem, some compensation can be made by reducing the water–binder ratio of concrete [36]. In the sulfate attack test, due to the addition of limestone powder, the content of gypsum in the erosion product is increased, which leads to the expansion and cracking of concrete, a decrease in strength, and a decrease in the sulfate attack resistance coefficient [37]. At the same water–binder ratio, the sulfate resistance of concrete decreases with the increase in limestone powder content [38].

The above studies have shown that the content of LP has a significant effect on the performance of concrete. However, the fineness of LP also has a significant impact on the performance of concrete [16]. Generally speaking, a higher fineness of LP results in a better filling effect. However, excessively fine powder can increase the viscosity of the concrete mixture, negatively impacting its workability [16,39]. In addition, when the particle size of LP is smaller than that of cement, it primarily serves a filling and chemical function. With the increase in LP content, the corrosion resistance of cement-based materials is improved. When the particle size of LP is comparable to that of cement, it primarily serves a dilutive function. With the increase in LP content, the corrosion resistance of steel bars decreases [39,40]. In summary, as a concrete admixture, LP can enhance the performance of concrete within a specific range; however, the effectiveness of this improvement is closely linked to the fineness of the LP. Currently, research in this area requires further enhancement, and a quantitative analysis of the relationship between its content, fineness, and water–binder ratio is necessary. Therefore, in this paper, 88 groups of LP concrete mix ratios were designed, using LP content, LP fineness, and the water–binder ratio as variables. The physical properties (slump) and mechanical properties (compressive strength and bond strength with steel reinforcement) of the concrete were systematically tested and analyzed theoretically. The purpose of this paper is to explore the influence of LP fineness and content on these properties, as well as the underlying patterns of change. At the same time, this paper proposes a compressive activity index (CAI) to evaluate and quantify the compressive activity of LP based on relevant literature. The aim is to provide a theoretical foundation for the application of LP in concrete.

## 2. Experimental Program

**Figure 1:**
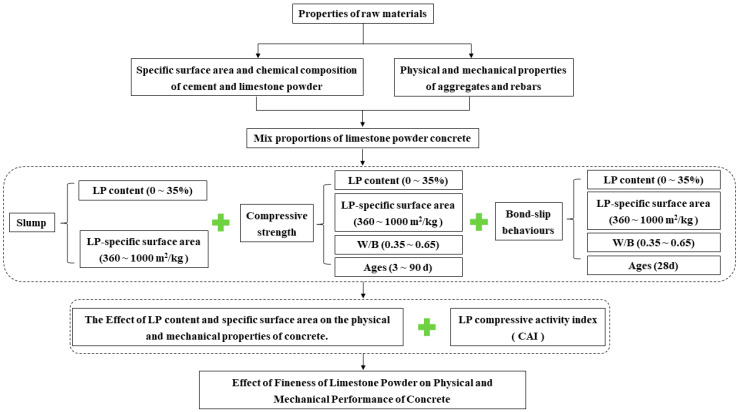
The technical route of the effect of limestone powder fineness on the physical and mechanical properties of concrete.

In the mixtures of LP concrete designed in this study, the 28-day compressive strength covers C20 to C60, which meets the general engineering requirements. In order to accurately explore the effect of LP fineness on the performance of concrete, no admixtures (such as water reducers) were added during the testing process. It is suggested that the influence of admixtures on the performance of LP concrete be considered in future applications and research. The technical route of the effect of limestone powder fineness on the physical and mechanical properties of concrete is depicted in Figure 1.

### 2.1. Materials

In the test, the cement, limestone powder, natural river sand, and gravel used were all from Jiangsu Zhuben Concrete Co., Ltd. (Xuzhou, China), and their properties are as follows:

(1) Cementitious material: P.O 52.5 cement, specific surface area of 300 m^2^/kg; limestone powder (LP), specific surface area of 350 m^2^/kg, 600 m^2^/kg, and 1000 m^2^/kg. The chemical composition of cement and LP is shown in Table 1.
(2) Fine aggregate: natural river sand; the fineness modulus is 2.68, the apparent density is 2630 kg/m^3^, and the mud content is 1.27%.
(3) Coarse aggregate: continuous graded crushed stone; the maximum particle size is 31.5 mm.
(4) Mixing water: commonly used tap water.
(5) Rebar: HRB400, diameter 16 mm; mechanical properties shown in Table 2.

**Table 1:** Chemical composition of cementitious materials (%).

Elements	CaO	SiO_2_	Al_2_O_3_	Fe_2_O_3_	K_2_O	Na_2_O	MgO	TiO_2_	SO_3_	MnO	Cl^−^	P	S
P.O 52.5	62.90	21.00	4.78	2.98	0.61	0.19	1.95	0.21	1.53	/	/	/	/
LP	51.00	2.27	0.46	0.24	0.18	/	1.95	<0.01	1.53	≤0.01	/	<0.01	0.09

**Table 2:** Mechanical properties of steel bars.

Rebar	Diameter (mm)	Length (mm)	Yield Strength (Mpa)	Ultimate Strength (Mpa)	Elongation (%)
HRB400	16	80	410	595	23.3

### 2.2. Mixture Proportions

In this paper, based on four groups of benchmark concrete mixtures, 84 groups of limestone powder concrete mixture proportions were designed (see Table 3 for details). Numbers were given as Xm-n, where the letter ‘X’ refers to A, B, C, and D, representing four 0.35, 0.45, 0.55, and 0.65 water–binder ratios; m represents limestone powder content (mass percentage), from 0% to 35%; and n represents the specific surface area of limestone powder, with 1, 2, and 3 corresponding to 350, 600, 1000 m^2^/kg. For example, A5-2 represents the concrete mix ratio with a water–binder ratio of 0.35, a limestone powder content of 5%, and a specific surface area of 600 m^2^/kg. It should be noted that Table 3 does not give the specific amount of cement and limestone powder in the mix ratio of limestone powder concrete, which needs to be calculated according to the amount of admixture.

### 2.3. Specimen Design

(1) Slump: The concrete mix ratios in Table 3 are sampled, and the slump test is carried out. The slump difference between limestone powder concrete and reference concrete is compared and analyzed.
(2) Compressive strength: According to the concrete mix ratio in Table 3, 150 mm cube specimens of 6 ages (3 d, 7 d, 14 d, 28 d, 56 d, and 90 d) were produced for a total of 528 groups (1584). The specimens were cured to the corresponding age for compressive strength test. The number of the specimen is the same as that of the concrete mix ratio.
(3) Bonding performance: (a) fixed water–binder ratio (W/B = 0.45) to study the effect of limestone powder content and specific surface area on the bonding performance. (b) Fixed limestone powder content (15%) and specific surface area (600 m^2^/kg); the effect of water–binder ratio on bonding performance was studied. (c) Select the B0 concrete mix ratio as the benchmark group. The detailed test scheme is shown in Table 4.

### 2.4. Test Methods

**Figure 2:**
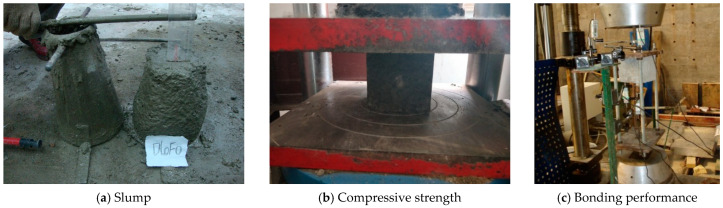
Test process.

The test was carried out according to the relevant literature [41,42,43,44], and the test process, as shown in Figure 2.

(1) Use a slump meter to measure the slump of concrete. The concrete is evenly loaded into the barrel in three layers, and each layer is evenly mashed 25 times. After the top layer is mashed, it is flattened with a spatula, and the excess part is removed. After the slump cylinder is lifted, the slump value is obtained by measuring the difference between the height of the cylinder and the highest point of the concrete slump.
(2) The compressive strength test was carried out by a YAW-3000 testing machine from Jinan Chenda Testing Machine Manufacturing Co., Ltd. (Jinan, China). After the specimen was wiped clean, it was placed in the center of the lower pressure plate, and the test machine was started and continuously and uniformly loaded until the specimen was sharply deformed and finally destroyed; the load value at this time was recorded.
(3) In the bond performance test, the continuous displacement loading speed of 0.2 mm/min is carried out. At the same time, the displacement meter is set at both ends of the steel bar, and the relative slip between the loading end and the free end and the concrete is recorded until the bond failure, and the slip is recorded throughout the process.

## 3. Results and Discussions

### 3.1. Slump

#### 3.1.1. LP Content

**Figure 3:**
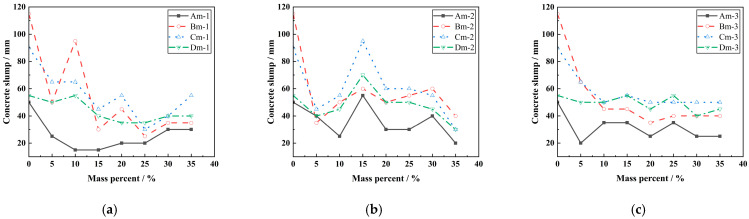
Effect of LP content on concrete slump. (**a**) The effect of LP with specific surface area of 350 m^2^/kg on the slump of concrete under different content. (**b**) The effect of LP with specific surface area of 600 m^2^/kg on the slump of concrete under different content. (**c**) The effect of LP with specific surface area of 1000 m^2^/kg on the slump of concrete under different content.

Figure 3 shows the effect of LP content on the concrete slump. It can be seen from Figure 3 that the slump of concrete mixed with LP is mostly smaller than that of reference concrete. With the increase in LP content, the slump of concrete sometimes shows a strengthening effect. As shown in Figure 3b, when the specific surface area of LP is 600 m^2^/kg, 15% content of LP has a certain improvement effect on the slump of concrete. At the same time, when the water–binder ratio is 0.35, 0.55, and 0.65, the slump of limestone powder concrete is higher than that of the reference concrete, and it has better plasticizing and pumpability. When the content of LP is the same, the change rule of the slump of limestone powder concrete is the same as that of ordinary concrete, which increases with the increase in the water–binder ratio.

#### 3.1.2. LP-Specific Surface Area

**Figure 4:**
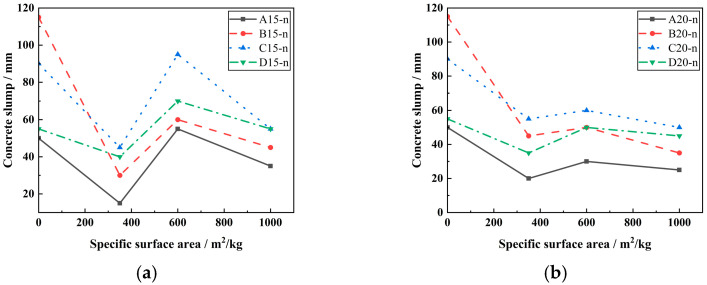
Effect of LP-specific surface area on concrete slump. (**a**) The effect of 15% content LP on the slump of concrete under different specific surface areas. (**b**) The effect of 20% content LP on the slump of concrete under different specific surface areas.

Figure 4 depicts the effect of LP-specific surface area on a concrete slump. It can be seen from Figure 4 that the slump of concrete mixed with LP is less than that of reference concrete. When the specific surface area of LP is 600 m^2^/kg, it has a certain improvement effect on the slump of concrete, but its slump value is still smaller than that of reference concrete. When the content of LP is large, as shown in Figure 4b, the slump of concrete changes gently within a certain range, indicating that the increase in LP-specific surface area has no obvious effect on the slump of concrete.

Comprehensive analysis shows that LP has a good filling effect and the ability to disperse cement particles, which can reduce the water consumption of concrete. Therefore, the proper addition of LP can improve the working performance of concrete and increase the slump. However, since the specific surface area of LP is larger than that of cement, as its content continues to increase, more water is needed to wet it, resulting in an increase in the water consumption of the mixture, which in turn reduces the slump of the concrete. This is similar to the conclusion in study [39].

### 3.2. Compressive Strength

#### 3.2.1. LP Content

**Figure 5:**
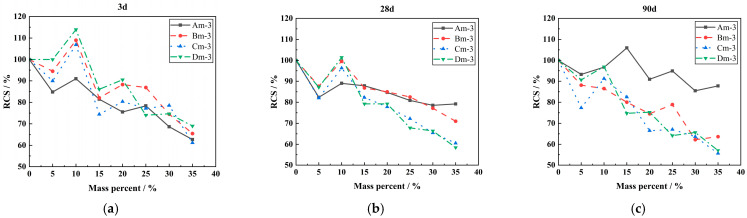
Effect of LP content of 1000 m^2^/kg on RCS of concrete. (**a**) The effect of LP with specific surface area of 1000 m^2^/kg on the RCS of concrete for 3 days under different content. (**b**) The effect of LP with specific surface area of 1000 m^2^/kg on the RCS of concrete for 28 days under different content. (**c**) The effect of LP with specific surface area of 1000 m^2^/kg on the RCS of concrete for 90 days under different content.

Figure 5 demonstrates the effect of the content of LP on the relative compressive strength (the ratio of the compressive strength of concrete to the compressive strength of the reference concrete at the same age, RCS) when the specific surface area of LP is 1000 m^2^/kg. As shown in Figure 5, the compressive strength of concrete decreases with an increase in LP content. However, when the content of LP is 10%, it has a clear strengthening effect on the compressive strength of concrete, which is higher than 80% of the reference concrete. The compressive strength of some limestone powder concrete was greater than that of benchmark concrete.

**Figure 6:**
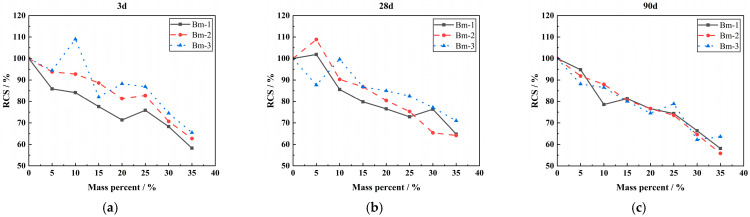
The effect of LP content on the RCS of concrete when the W/B ratio is 0.45. (**a**) When the W/B ratio is 0.45, the effect of LP on the RCS of concrete for 3 days under different content. (**b**) When the W/B ratio is 0.45, the effect of LP on the RCS of concrete for 28 days under different content. (**c**) When the W/B ratio is 0.45, the effect of LP on the RCS of concrete for 90 days under different contents.

Figure 6 presents the effect of LP content on the RCS of concrete when the W/B ratio is 0.45. As shown in Figure 6, when the W/B ratio is fixed, the compressive strength of concrete decreases with the increase in LP content. Before 28 days, when the content of LP is less than 10%, the compressive strength of concrete is maintained at more than 80% of the reference concrete, and some specimens even exceed the reference concrete. It shows that LP has a strengthening effect on the compressive strength of concrete, which is consistent with the results obtained in Figure 5.

#### 3.2.2. LP-Specific Surface Area

**Figure 7:**
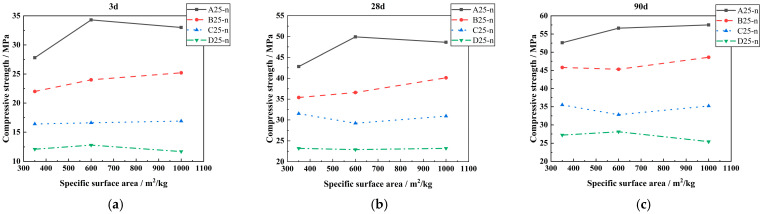
Effect of LP-specific surface area on compressive strength of concrete. (**a**) The effect of 25% content LP on the 3-day compressive strength of concrete under different specific surface areas. (**b**) The effect of 25% content LP on the 28-day compressive strength of concrete under different specific surface areas. (**c**) The effect of 25% content LP on the 90-day compressive strength of concrete under different specific surface areas.

Figure 7 displays the effect of specific surface area on the compressive strength of concrete when the content of LP is 25%. It can be seen from Figure 7 that with the increase in LP-specific surface area, the compressive strength of concrete increases slightly. It shows that the effect of increasing the specific surface area of LP on the compressive strength of concrete is not obvious when LP is more than 15% and no admixture is added.

#### 3.2.3. W/B

**Figure 8:**
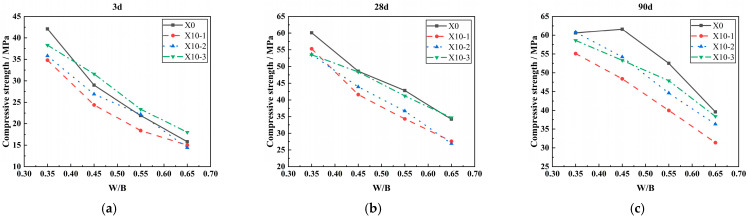
Effect of the W/B ratio on compressive strength of concrete. (**a**) The effect of 10% content LP on the 3-day compressive strength of concrete under different W/B ratios. (**b**) The effect of 10% content LP on the 28-day compressive strength of concrete under different W/B ratios. (**c**) The effect of 10% content LP on the 90-day compressive strength of concrete under different W/B ratios.

Figure 8 illustrates the effect of the W/B ratio on the compressive strength of concrete when the LP content is 10%. As shown in Figure 8, the compressive strength of limestone powder concrete decreases with the increase in the W/B ratio. At the age of 28d, when the specific surface area of LP increased from 350 to 1000 m^2^/kg, the compressive strength of concrete with 0.65 water–binder ratio decreased by 50.1%, 49.8%, and 35.1%, respectively, compared with that of concrete with 0.35 water–binder ratio. This shows that the finer the LP particles, the better the filling effect, and the smaller the effect of the W/B ratio on the decrease in compressive strength of concrete [39].

#### 3.2.4. Ages

**Figure 9:**
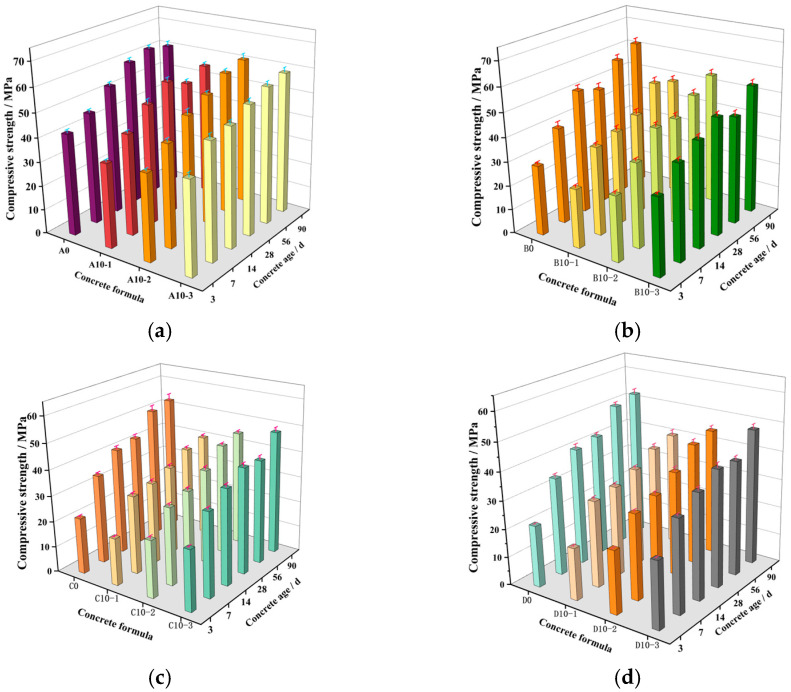
Effect of age on compressive strength of concrete. (**a**) When the W/B ratio is 0.35, the effect of 10% content LP on the compressive strength of concrete at different ages. (**b**) When the W/B ratio is 0.45, the effect of 10% content LP on the compressive strength of concrete at different ages. (**c**) When the W/B ratio is 0.55, the effect of 10% content LP on the compressive strength of concrete at different ages. (**d**) When the W/B ratio is 0.65, the effect of 10% content LP on the compressive strength of concrete at different ages.

Figure 9 reveals the effect of age on the compressive strength of concrete when the LP content is 10%. It can be seen from Figure 9 that the compressive strength of limestone powder concrete increases with the increase in age, the growth rate is faster in the early stage, and the speed slows down after 60 days. At the same age, the compressive strength of limestone powder concrete increases with the increase in LP-specific surface area.

### 3.3. Bond-Slip Behaviors

#### 3.3.1. Failure Morphology

**Figure 10:**
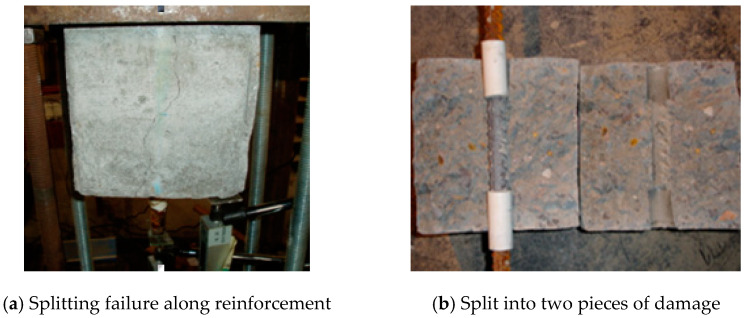
Failure morphology of the specimen.

The brittle failure occurred in all bonding specimens in this test. Because there is no stirrup in the specimen, when the stress reaches the peak value, the specimen will quickly split along the reinforcement, and some specimens will even split into two instantaneously, accompanied by a crisp splitting sound, as shown in Figure 10.

#### 3.3.2. Bonding Strength

The bonding strength when the bond specimen is destroyed is shown in Table 5.

According to the data in Table 5, when the content of LP increases from 5% to 25%, the bond strength between limestone powder concrete and steel bar decreases by 1.58%, 10.09%, 20.38%, and 39.88%, respectively, compared with the reference specimen. With the increase in LP content, the decreased rate of bond strength becomes larger. However, when the LP content is less than 15%, the reduction in bond strength is less than 10%, so it is recommended to control the LP content within 15%. At the same time, when the specific surface area of LP increases from 350 m^2^/kg to 1000 m^2^/kg, the bond strength decreases by 47.19%, 13.82%, and 10.16%, respectively, compared with the reference specimen, indicating that increasing the specific surface area of LP is helpful to enhance the bond strength. In addition, when the W/B ratio is 0.35 and 0.45, the bond strength is increased by 20.35% and 7.05%, respectively, compared with the specimen with a W/B ratio of 0.55, which is consistent with the influence trend of LP on concrete strength.

#### 3.3.3. Effect of LP Content on *τ*-*s* Curves

**Figure 11:**
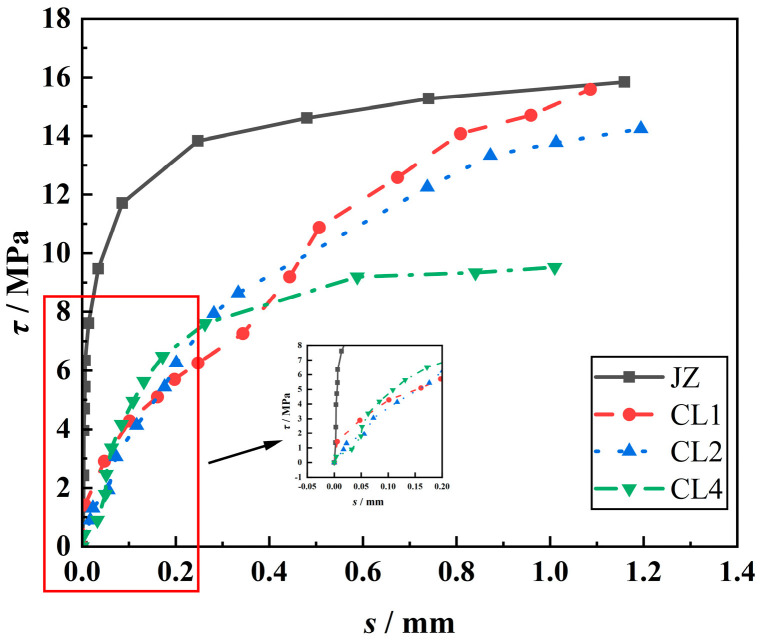
Effect of LP content on the *τ*-*s* curves of the specimens.

Figure 11 shows the effect of LP content on the *τ*-*s* curves of the specimens. It can be seen from Figure 11 that the average bond stress (*τ*) of the steel–concrete bond specimens with LP is lower than that of the reference specimens and decreases with the increase in LP content. Under the same conditions, the corresponding bond stress of the reference specimen is the largest when the free end begins to slide, followed by the specimen with 5% LP content. Under the same bond stress, the free end slip (*s*) of the reference specimen is the smallest, and the bond stress is the largest when it is destroyed. With the increase in LP content, the slip of the free end increases. This is because the appropriate amount of LP can improve the internal structure of concrete and improve the strength, with the ‘filling and nucleation’ effect; however, excessive incorporation of LP will reduce cement clinker, reduce hydration products and strength, resulting in a decrease in bond strength and an increase in free-end slip [39,45].

#### 3.3.4. Effect of LP-Specific Surface Area on *τ*-*s* Curves

**Figure 12:**
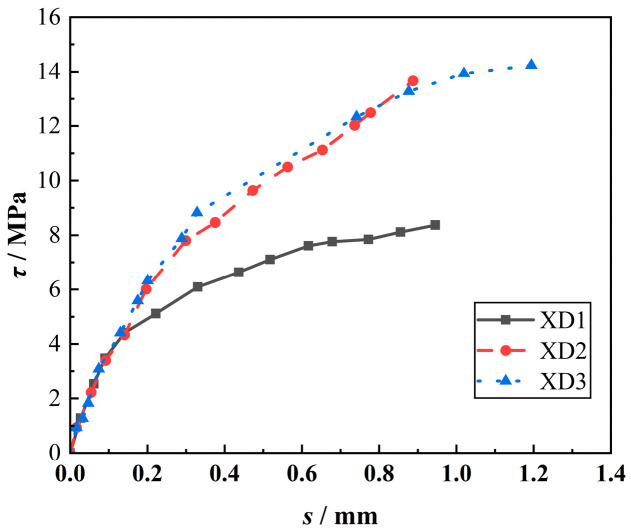
Effect of LP-specific surface area on the *τ*-*s* curves of the specimens.

Figure 12 shows the effect of LP-specific surface area on the *τ*-*s* curves of the specimens. It can be seen from Figure 12 that when *τ* is greater than 5 MPa, the *τ* of the specimen increases with the increase in LP-specific surface area. At the same *τ*, the free end slip (*s*) decreases with the increase in LP-specific surface area. Especially when the specific surface area of LP increased from 350 m^2^/kg to 600 m^2^/kg, *s* decreased rapidly, and then the change tended to be stable. This is because with the increase in the specific surface area of LP, its ‘filling and nucleation’ effect is better played, and the effect is the best at 600 m^2^/kg, thereby enhancing the strength and *τ* of concrete and reducing the free end slip. However, when the specific surface area of LP exceeds 600 m^2^/kg, the effect on the strength of concrete is weakened, so *τ* only increases slightly.

#### 3.3.5. Effect of W/B on *τ*-*s* Curves

**Figure 13:**
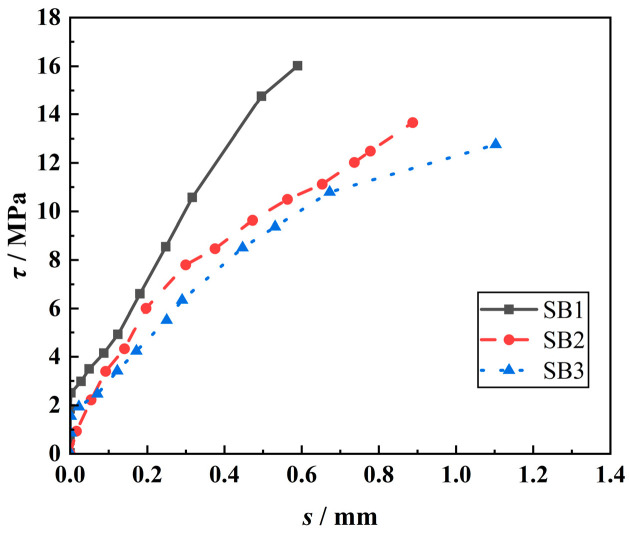
Effect of the W/B ratio on the *τ*-*s* curves of the specimens.

Figure 13 shows the effect of the W/B ratio on the *τ*-*s* curves of the specimens. It can be seen from Figure 13 that when the LP content and the specific surface area are the same, the bond strength at the failure of the bond specimen is inversely proportional to the W/B ratio, while the free end slip is proportional. With the increase in the W/B ratio from 0.35 to 0.45, the bond strength of the bonded specimens decreased by 1.07%. Therefore, in the case of adding LP, a suitable water-reducing agent can be found to reduce the water–binder ratio of concrete, thereby increasing the bond stress between concrete and steel bars. This indicates the research direction and significance for the application of LP in concrete.

## 4. Compressive Activity of LP

**Figure 14:**
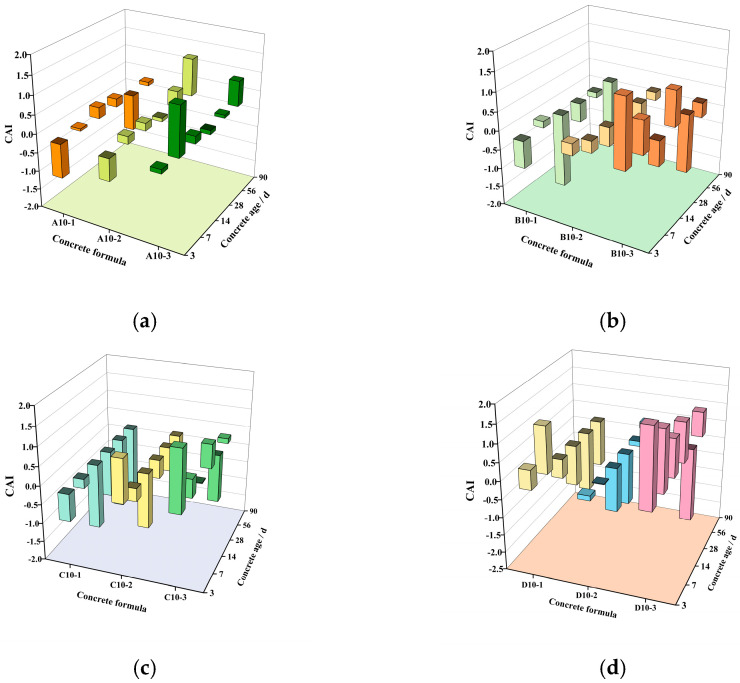
The 10% content of LP compressive activity index. (**a**) When W/B is 0.35, the CAI of LP with 10% content at different ages. (**b**) When W/B is 0.45, the CAI of LP with 10% content at different ages. (**c**) When W/B is 0.55, the CAI of LP with 10% content at different ages. (**d**) When W/B is 0.65, the CAI of LP with 10% content at different ages.

The compressive activity index (CAI) was established to assess the compressive activity of LP in conjunction with the relevant literature [46,47]. The index is computed using Equations (1)–(5), and the outcomes are displayed in Figure 14.

(1) $$R_{L}^{\prime }=\frac{R_{L}}{Q_{C}}$$

(2) $$R_{C}^{\prime }=\frac{R_{C}}{Q_{C}}$$

(3) $$R_{H}=R_{L}^{\prime }-R_{C}^{\prime }$$

(4) $$P_{H}=\frac{R_{H}}{R_{L}^{\prime }}\times 100\%$$

(5) $$CAI=\frac{P_{H}}{Q_{L}}$$

where $R_{L}^{\prime }$ is the specific strength of limestone powder concrete, and the unit is MPa; *R_L_* is the compressive strength of limestone powder concrete in MPa; *Q_C_* is the proportion of cement in cementitious materials, %; $R_{C}^{\prime }$ is the specific strength of the reference concrete, and the unit is MPa; *R_C_* is the compressive strength of the reference concrete in MPa; *R_H_* is the specific strength of the active effect of limestone powder concrete, and the unit is MPa; *P_H_* is the contribution rate of activity effect intensity of LP, %; *CAI* is the compressive activity index of LP; *Q_L_* is the proportion of LP in cementitious materials, %.

Figure 14 shows the compressive activity index of LP with 10% content. It can be seen from Figure 14 that under the same conditions, the compressive activity index of LP increases with the increase in specific surface area and decreases with the increase in age, which is consistent with the previous results. In addition, it should be noted that LP is an inert material, and its pozzolanic activity effect is extremely low. The compressive activity index (CAI) proposed here refers to the filling effect, nucleation effect, and chemical effect of LP. The CAI is negative, indicating that LP plays a role in reducing the strength of concrete.

## 5. Conclusions

In this paper, experimental and theoretical studies were conducted to investigate the influence of LP content, LP-specific surface area, and water–binder ratio on the physical and mechanical properties of LP concrete. The CAI was proposed to evaluate and quantify the compressive activity of LP. The main conclusions are as follows:

(1) Adding the optimal quantity of LP can improve the working performance of concrete. However, when the specific surface area of LP surpasses that of cement, augmenting the LP content would result in an elevation in water requirement, therefore diminishing the slump of concrete. Specifically, the optimal improvement in the slump of concrete occurs when the specific surface area of LP is 600 m^2^/kg and its content is 15%.
(2) When the LP content is less than 15%, it can substantially enhance the compressive strength of concrete. In addition, LP with a specific surface area of 1000 m^2^/kg most improves concrete strength in the early stage, while limestone powder with a specific surface area of 600 m^2^/kg is optimal for improving strength in the later stage.
(3) The variation trend of the *τ*-*s* curves of steel bar–LP concrete is similar to that of steel bar–ordinary concrete. However, the ultimate bond stress is inversely proportional to the content of LP and W/B and is proportional to the specific surface area of LP.
(4) As an inert material, LP has a very low pozzolanic activity effect. The compressive activity index (CAI) proposed in this paper can effectively characterize this characteristic. CAI increases with the increase in LP-specific surface area and decreases with age. When CAI is negative, it indicates that LP reduces the strength of concrete.
(5) LP can be used as a preferred alternative when traditional mineral admixtures (such as fly ash and slag) are in short supply or expensive. When the specific surface area of LP is 600 m^2^/kg, and the content is not more than 15%, the substitution effect is the best, which can significantly improve the physical and mechanical properties of concrete. In view of the limitations of the test, the follow-up study needs to further explore the coupling effect of LP and fly ash and slag on the performance of concrete.

## Figures and Tables

**Table 3:** Mixture proportions of limestone powder concrete.

No.	LP Content (%)	LP Specific Surface Area (m^2^/kg)	W/B	Material Consumption Per Unit Volume (kg/m^3^)					*f*_cu_ (Mpa)	Quantities
				CE	LP	NS	CS	W		
A0	0	/	0.35	557	0	470	1174	195	60.1	1
A5/10/15/20/25/30/35-1/2/3	5/10/15/20/25/30/35	350/600/1000	0.35	/	/	470	1174	195	/	21
B0	0	/	0.45	433	0	563	1187	195	48.6	1
B5/10/15/20/25/30/35-1/2/3	5/10/15/20/25/30/35	350/600/1000	0.45	/	/	563	1187	195	/	21
C0	0	/	0.55	355	0	641	1175	195	42.8	1
C5/10/15/20/25/30/35-1/2/3	5/10/15/20/25/30/35	350/600/1000	0.55	/	/	641	1175	195	/	21
D0	0	/	0.65	300	0	711	1152	195	34.2	1
D5/10/15/20/25/30/35-1/2/3	5/10/15/20/25/30/35	350/600/1000	0.65	/	/	711	1152	195	/	21

Notes: LP stands for limestone powder; W/B denotes the water–binder ratio; CE represents cement; NS indicates natural sand; CS refers to crushed stone; W signifies the mixing water; and *f*_cu_ refers to the 28-day cube compressive strength of concrete.

**Table 4:** Bonding performance test scheme.

Specimen Types	Specimen No.	Formulation No.	Variable	LP Content (%)	LP Specific Surface Area (m^2^/kg)	Strength Grades	*c* (mm)	*l*_a_ (mm)	*d* (mm)	Quantities
CL	CL1	B5-3	LP content	5%	1000	C30	67	80	16	3
	CL2	B15-3		15%	1000	C30	67	80	16	3
	CL3	B20-3		20%	1000	C30	67	80	16	3
	CL4	B25-3		25%	1000	C30	67	80	16	3
XD	XD1	B15-1	LP-specific surface area	15%	350	C30	67	80	16	3
	XD2	B15-2		15%	600	C30	67	80	16	3
	XD3	B15-3		15%	1000	C30	67	80	16	/
SB	SB1	A15-2	W/B	15%	600	C40	67	80	16	3
	SB2	B15-2		15%	600	C30	67	80	16	/
	SB3	C15-2		15%	600	C20	67	80	16	3
JZ	JZ1	B0	/	0	/	C40	67	80	16	3

Note: CL, XD, SB, and JZ represent the groups of adjustment parameters, whereas CL2 and XD3 and XD2 and SB2 are actually the same group. Here, for ease of research, it is expressed separately. *c* refers to the thickness of the protective layer of concrete, *l*_a_ refers to the anchorage length of the steel bar, and *d* refers to the diameter of the steel bar.

**Table 5:** Bonding strength of bond specimens at failure.

Specimen Types	Specimen No.	W/B	LP Content (%)	LP Specific Surface Area (m^2^/kg)	Bonding Strength (MPa)
CL	CL1	0.45	5	1000	15.60
	CL2	0.45	15	1000	14.24
	CL3	0.45	20	1000	12.62
	CL4	0.45	25	1000	9.53
XD	XD1	0.45	15	350	8.37
	XD2	0.45	15	600	13.66
	XD3(CL2)	0.45	15	1000	14.24
SB	SB1	0.35	15	600	16.02
	SB2(XD2)	0.45	15	600	13.66
	SB3	0.55	15	600	12.76
JZ	JZ1	0.45	0	/	15.85

## Data Availability

The original contributions presented in this study are included in the article. Further inquiries can be directed to the corresponding authors.
